# Radiological safety status and quality assurance audit of medical X-ray diagnostic installations in India

**DOI:** 10.4103/0971-6203.71764

**Published:** 2010

**Authors:** A. U. Sonawane, Meghraj Singh, J. V. K. Sunil Kumar, Arti Kulkarni, V. K. Shirva, A. S. Pradhan

**Affiliations:** Atomic Energy Regulatory Board, India; 1 Ex- Atomic Energy Regulatory Board, Mumbai, India; 2 Ex-Bhabha Atomic Research Centre, Mumbai, India

**Keywords:** Diagnostic installations, quality assurance tests, safety audit

## Abstract

We conducted a radiological safety and quality assurance (QA) audit of 118 medical X-ray diagnostic machines installed in 45 major hospitals in India. The main objective of the audit was to verify compliance with the regulatory requirements stipulated by the national regulatory body. The audit mainly covered accuracy check of accelerating potential (kVp), linearity of tube current (mA station) and timer, congruence of radiation and optical field, and total filtration; in addition, we also reviewed medical X-ray diagnostic installations with reference to room layout of X-ray machines and conduct of radiological protection survey. A QA kit consisting of a kVp Test-O-Meter (ToM) (Model RAD/FLU-9001), dose Test-O-Meter (ToM) (Model 6001), ionization chamber-based radiation survey meter model Gun Monitor and other standard accessories were used for the required measurements. The important areas where there was noncompliance with the national safety code were: inaccuracy of kVp calibration (23%), lack of congruence of radiation and optical field (23%), nonlinearity of mA station (16%) and timer (9%), improper collimator/diaphragm (19.6%), faulty adjustor knob for alignment of field size (4%), nonavailability of warning light (red light) at the entrance of the X-ray room (29%), and use of mobile protective barriers without lead glass viewing window (14%). The present study on the radiological safety status of diagnostic X-ray installations may be a reasonably good representation of the situation in the country as a whole. The study contributes significantly to the improvement of radiological safety by the way of the steps already taken and by providing a vital feed back to the national regulatory body.

## Introduction

Medical X-ray diagnostic radiology is the largest contributor to public exposure to man-made ionizing radiation. About 80% of the dose to the population is estimated to be caused by medical diagnostic X-ray examinations.[1] The International Commission on Radiological Protection (ICRP) has stressed that all medical exposures should be guided by the radiation safety principles of justification and optimization.[2] The International Basic Safety Standards for Protection against Ionizing Radiation and for the Safety of Radiation Sources, Safety Series No.115, published by the International Atomic Energy Agency (IAEA-BSS), 1996,[3] recommends various technical, scientific, and administrative requirements for ensuring the protection of people from exposure to ionizing radiation and the safety of radiation sources. The requirements, *inter alia*, include the establishment and continued maintenance of a safety culture, quality assurance programme (QAP), monitoring and verification of compliance with safety requirements, safety assessment and verification of safety records, inspections, enforcement of appropriate actions in case of noncompliances, etc. Such requirements are also emphasized by several other international radiation protection organizations. These basic arrangements are referred to as ‘infrastructure for safety,’ and include laws and regulations on the safe use of radiation sources. The periodic conduct of safety audits are therefore essential to ensure that adequate ‘infrastructure of safety’ is available and effectively implemented by users of radiation. Some studies on safety audits have been carried out earlier to assess the radiological safety status of diagnostic X-ray installations in the country.[4–7]

In India, nearly 1000 new units begin operating annually and currently there are more than 50000 medical diagnostic X-ray units[8] functioning all over the country. The Atomic Energy Regulatory Board (AERB) of India is the national regulatory body and the competent authority for enforcing regulatory provisions for radiation protection in India.[9] The AERB is responsible for laying down the national safety codes and standards that stipulate the requirements for safe handling of radiation sources, including medical diagnostic X-ray installations.[10] The AERB has also published the National Safety Code on Medical Diagnostic X-ray Equipment and Installations, No. AERB/SC/MED-2,Rev.01, (2001).[11] The safety code[11] is intended to govern radiation safety in design, installation, and operation of X-ray equipment for medical diagnostic purposes. The implementation of the provisions of the safety code ensures the protection from radiation of occupational workers, patients, and the public. Some of the regulatory requirements of the safety code[11] are as follows:

1. Only those X-ray machines should be procured by the users that have been type approved by the competent authority.
2. Installation and room layout should be in accordance with the specifications of the safety code.
3. On acquisition (by purchase, transfer, gift, lease, or loan) of the X-ray equipment the user should ensure that it is registered with the competent authority.
4. The Radiological Safety Officer (RSO) should conduct periodic radiological protection surveys of the X-ray installation and maintain records of routine QA tests on the X-ray machine.
5. All radiation workers should use appropriate personnel monitoring badges (PMB).

The safety code emphasizes that the ultimate responsibility of ensuring radiation safety, availability of RSO and qualified personnel for handling of X-ray equipment rests with the employer.

It is generally presumed that the users take a due note of the requirements of the safety code and a comprehensive QA tests for every X-ray machine are carried out at the time of installation and thereafter as per the requirements of the national safety code. The main objective of the present work was to carry out an independent audit of the important features of QA tests and radiation safety parameters in major medical X-ray installations, which would reflect the safety status in the country, and to assess compliance with the regulatory requirements of the safety code.

## Materials and Methods

**Figure 1:**
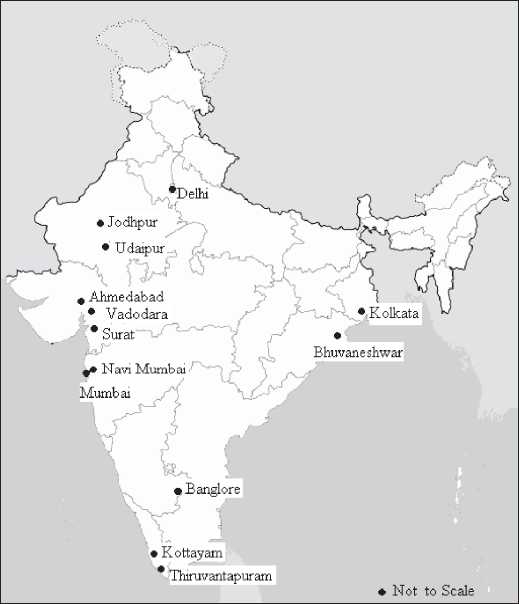
Radiological safety and QA audit carried out at various diagnostic X-ray installations in India

A large number of hospitals belonging to the government sector, the private sector, and public trusts were approached for inclusion in the study. From among those who readily responded, extended cooperation, and were conveniently accessible to the authors, 118 medical diagnostic X-ray machines installed in 45 major hospitals located in 13 cities in India [Figure 1] were selected for the present study. We feel that this should reflect the countrywide scenario. The audit included major QA tests on every X-ray machine, conduct of radiological protection surveys at each X-ray installation, and verification of the implementation of the regulatory requirements of the safety code.[11] Table 1 gives the list of models/manufacturers of diagnostic medical X-ray machines – with the important technical specifications [phase/ type of rectification, total filtration, maximum ratings (kVp and mA)] – covered during this study.

**Table 1:** The model/manufacturer and major technical specifications of medical diagnostic X-ray machines (phase/type of rectification, total filtration, maximum ratings (kVp and mA)

*Model*	*Technical specifications*			
	*Phase/ rectification*	*Total filtration (Al)*	*Max. kVp*	*Max. mA*
Allenges-525	Single	3 mm	125	500
Allenges-525	Three	3 mm	125	300
DSA-3, Elpro	Single	2.5 mm	100	100
DX-300	Single	3.5 mm	100	300
DX-300/350	Single	3 mm	100	350
DX-525	Single	3 mm	125	500
DXD-300, Elpro	Single	3 mm	100	300
DXD-300, Elpro	Single	3.5 mm	100	300
Electromedical	Three	2.5mm	125	500
Electronet	Three	2.5mm	125	500
ElproD	Single	3 mm	100	300
EMAI	Single	3 mm	150	300
Ergoplus 4M	Single	3 mm	100	300
Genius-60	Single	2mm	100	60
Heliophos D	Single	3 mm	125	500
Heliophos D	Single	3 mm	125	300
Heliophos D	Three	3 mm	125	500
Heliophos D	Single	3 mm	125	300
Medex /Mediray	Three	2.5 mm	100	100
Meditronix Diagnox 300	Single	3 mm	100	300
Meditronix	Single	3 mm	125	300
Pleophos-D	Single	3 mm	125	300
Pleophos-D	Three	3 mm	125	300
Pleophos D	Three	4 mm	125	300
Polymar-50 I	Three	3 mm	100	500/800
Polyscope-II	Single	3 mm	100	300
Siemens GT-107	Three	4 mm	125	300
Siemens Optix /polydors	Three	4 mm	150	800
Siemens T-6R	Three	4 mm	150	300
Simplex/DX-300	Single	3 mm	125	300
SRD 3D/300	Single	3.5 mm	100	300
SRD-300	Single	3.5 mm	100	300
SRD-725	Single	3.5 mm	125	700
Stallion-20	Single	2 mm	85	20
Stallion 60	Single	2 mm	85	60

A QA kit (M/s UNFORS, Sweden) consisting of state-of-the-art instruments, viz., Test-O-Meter (ToM) (Model RAD/FLU-9001) for kVp measurement and the solid-state detector–based dose Test-O-Meter (Model 6001) for air kerma measurement were used. These instruments provided instantaneous and reliable values. The kVp meter automatically measures kVp in the range from 55 kVp to 145 kVp with a resolution of 0.1 kV, providing the results within seconds and updating it every second for fluoroscopy. The dose ToM incorporates a sealed silicon detector having a lead shield under and around, which prevents backscattered radiation from influencing the measurement. The ToMs have excellent energy-independent response in the range from 50 kVp to 150 kVp and inaccuracy not exceeding 5% at 70 kVp. The ToMs for kVp and dose measurements were tested and calibrated at the national secondary standard laboratory at the Bhabha Atomic Research Centre (BARC), Mumbai. The calibration of ToMs was traceable to the National Secondary Standard Laboratory (RSSD, BARC) and also to the National Institute of Standards and Technology (NIST, USA) and Physikalisch-Technische Bundesanstalt (PTB, Germany) as per the certificates provided by the supplier. The uncertainty in the measurement of kVp and air kerma was found to be within ± 2% and ± 5%, respectively. This was verified by using two standard dosimeters, viz., free-in-air PTW-IC, 0.3 cc chamber and the Victoreen model 8000, NEROmAx, and by using an X-ray machine (model Polydoros-LX of Siemens), the output consistency of which was verified separately.[12] The response of three reference dosimeters was checked after verifying the accuracy of the radiation parameters of the diagnostic X-ray machine model Polydoros-LX and other QA parameters. The accuracy of the operating potential (kV) was found to be within ± 3 kV and the coefficient of variation (s/m) was found to be less than 0.003.[13]

The radiological protection surveys were carried out by using 400 cc indigenously manufactured ionization chamber–based radiation survey meter model Gun Monitor (GM-125), with its calibration also traceable to the National Secondary Standard Laboratory.

**Figure 2:**
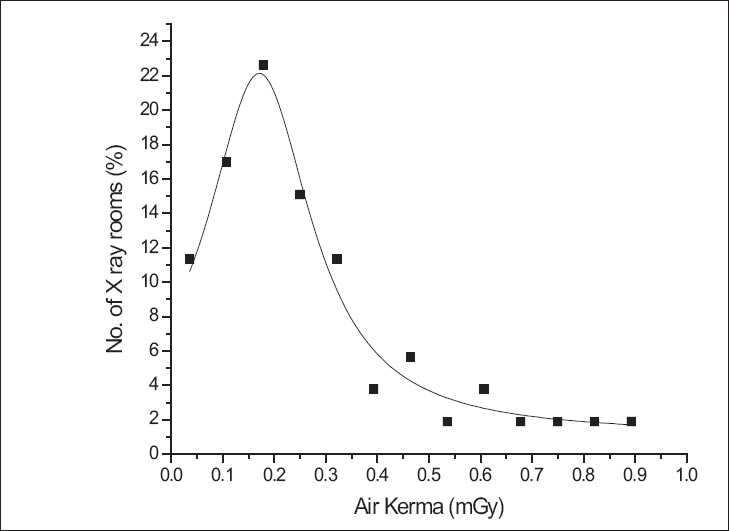
Number of X-ray rooms and variation in output of X-ray machines (air kerma - free in air) (TDD = 150 cm, mean kVp = 58, mean mAs = 14)

Table 2 gives the acceptance criteria[14] of major QA tests that were performed on various X-ray machines. For the verification of the accelerating potential (kVp), tube current values from 15 mAs to 20 mAs and target-to-detector distance (TDD) of 50 cm were used. The kVp ranges verified include kVp values from 55 kVp to 100 kVp (i.e., 60, 70, 80/81, 90, and 100). The difference between the kVp set on the control panel (CP) and measured by RAD/FLU kVp meter was determined for ensuring the acceptance criteria of ±5 kVp. For assessing the linearity of tube current (mA station), TDD of 100 cm, accelerating potential from 70 kVp to 80 kVp, and exposure time of 0.1 second were used. For assessing the linearity of timer, TDD of 100 cm, accelerating potential from 60 kVp to 70 kVp, and tube current of 100 mA to 150 mA were used. The air kerma was measured at different mAs values and the ratio of air kerma to mAs was obtained to determine the coefficient of linearity (CoL) to verify the compliance with the acceptance criteria (CoL < 0.1). For testing the congruence of radiation and optical field we used the congruence tool manufactured by BARC; a photographic film of 10 inches × 12 inches was exposed at target-to-film distance (TFD) of 100 cm for exposure settings 10 mAs to 12 mAs at 60 kVp. The total filtration of the X-ray tube was verified by estimating the Half-Value Layer (HVL). For this purpose, a dose ToM (model 6001) and five aluminium (Al) filters (sheets) of thickness 1 mm, 2 mm, 3 mm, 4 mm, and 5 mm, respectively, were used. Dose ToM was kept at 100 cm from the X-ray target and the radiation field collimated to dose ToM size. The first exposure was made at frequently used kVp and about 30 mAs without any filter interposed. Then, an Al filter of 1 mm was placed over the dose ToM and the exposure was measured. Subsequently, the thickness of the Al filter was increased in steps from 1 mm to 5 mm. Each time, three measurements of exposure were taken. After completion of measurements, a curve was plotted with the *μ*Gy/mAs reading on the Y-axis and the Al filter thickness in mm on the X-axis. Half value layer (HVL) of the X-ray beam at the given kVp was evaluated in terms of Al thickness in mm from the graph. The total tube filtration was determined based on the available nomograms showing the relation between HVL and total filtration of the X-ray beam. A minimum total filtration of at least 2.5 mm of Al was observed in all the cases. The output of X-ray machines in terms of values of air kerma-free in air at 150 cm TDD, 58 kVp (mean value), and 14 mAs (mean value) is shown in Figure 2.[12]

**Table 2:** The major QA tests and acceptance criteria for medical X-ray diagnostic machines[14]

*QA test*	*Acceptance criteria*
Congruence of radiation and optical field	Shift in the edges of radiation field within 2% of TFD Differences in dimensions of radiation and optical field within 3% of TFD Differences of sum of lengths and width of radiation and optical field within 4% of TFD
Accuracy of accelerating	within ±5 kVp
potential (kVp)	
Linearity of mA station	Coefficient of linearity < 0.1
Linearity of timer	Coefficient of linearity < 0.1
Output consistency	Coefficient of variation (CoV) < 0.05
Total tube filtration	> 2.5 mm of Al

The stray radiation levels at different locations in and around the X-ray room were measured by setting maximum kVp and minimum mA with more than 1.5-second exposure time. A water phantom placed at 100 cm of TFD was used to represent a patient. A rough sketch of the layout of the X-ray installation was drawn. The sketch indicated the dimensions of the room and the location of the X-ray tube, the control panel, the personnel entrance door (PED), the dark room, and the generator; the nature of occupancy around the installation was also shown. The technical specifications of the X-ray machine, the layout of the X-ray room, the availability of personnel monitoring badges, etc., were also verified. The stray radiation levels were measured at the following locations:

- Operator position at the control panel and behind mobile protective barrier
- Outside the mobile protective barrier
- Radiation outside the personnel entrance door (PED) and windows and other occupied areas.
- Cable crossings, opening in walls, and other areas behind the chest stand.
- Occupied areas in and around the installations.

Variations in stray radiation levels (minimum to maximum) observed during the study are shown below:

- At control room: 1–15 mR/h
- At PED: 4–800 mR/h
- Patient waiting area: 0.8–12 mR/h
- Behind mobile protective barrier: 2–60 mR/h
- Outside mobile protective barrier: 25–1500 mR/h

## Results and Discussion

The radiological safety and QA audits were carried out by performing major QA tests on 118 medical diagnostic X-ray machines installed in 45 major hospitals in 13 cities in India [Figure 1], thus covering almost all regions of the country. The study covered about 27 different models of X-ray machines. Most of the machines had mA station up to 300 mA. The QA tests revealed that out of 118 X-ray machines, 27 did not meet the acceptance criteria for congruence of radiation and optical field, while a need for calibration was observed in 28 machines for kVp, in 19 for mA, and in 11 for timer.

Table 3 shows the percentage of X-ray rooms and X-ray machines not complying with the requirements of radiological safety and QA parameters as per the national safety code.[11] Some of the observed major noncompliances were with regard to: inaccuracy of kVp calibration (23%); noncongruence of radiation and optical field (23%); nonlinearity of mA station (16%) and timer (9%); faulty adjustor knob for alignment of field size (4%); nonavailability of warning light (red light) at the entrance of the X-ray room (29%); having mobile protective barriers without lead glass viewing window (14%); improper collimator/diaphragm (19.6%), etc. The observed noncompliances were communicated to the concerned hospital so that immediate corrective actions could be initiated. The manufacturer/supplier of the X-ray machines were consulted by the concerned hospital to rectify the technical problems of the X-ray machine relating to accelerating potential (kVp), mA station, timer and alignment of radiation, and optical field and replacement of the faulty field size adjuster knobs. Four hospitals discarded the faulty X-ray machines. With regard to radiation safety parameters such as provision of warning light (red light) at the entrance of X-ray room; replacement of bulb inside the collimator; and making modifications in the layout of the X-ray room to relocate the chest stand, control panel, patient waiting areas, dark room, and mobile protective barriers, etc., from a radiological safety viewpoint were corrected during the course of the visit to the hospital for this study. The replacement/provision of mobile protective barriers with a lead glass viewing window and availability of personnel monitoring badges were confirmed by the hospitals subsequently.

**Table 3:** Percentage of X-ray rooms and X-ray machines not complying with requirements of radiological safety and QA parameters as per the National Safety Code[11]

*Noncompliances with radiological safety requirements*	*% of X-ray rooms*
Warning light (red light) not provided outside X-ray room	28.57
Warning light (red light) provided but not used	19.64
Personal monitoring badge (PMB) not provided to staff in radiology department	14.29
PMB not used	12.50
Location of chest stand near personal entrance door (PED)	12.50
Location of chest stand near window	5.36
Location of dark room not OK	5.36
Collimator/diaphragm not satisfactory	19.60
Location of control panel not OK	5.36
Mobile protective barrier not used	14.29
Mobile protective barrier without lead glass viewing window	14.29
Patient waiting area not satisfactory	7.14
Room size not adequate	7.14
Auto ON/OFF switch of control panel not working	7.14
Bulb in the collimator /diaphragm not working	12.50
Field size adjuster knob not functioning	3.57
Qualified personnel not available	8.93
PED needing lead lining	19.64
*Noncompliances with QA parameters*N	*% of X-ray machines*
Noncongruence of radiation and optical field	23.21
kVp needing calibration	23.21
mA station needing calibration	16.07
Timer needing calibration	8.93

In view of the observed noncompliances with requirements of the safety code and in the interests of radiation protection of persons in working in diagnostic radiology, we suggest that a suitable method be established in the country for verification of effective adherence with the provisions of the national safety code by the X-ray departments of hospitals. For this purpose, it is necessary to establish a Radiation Safety Directorate/ Agency for medical X-ray practice in every state of the country and also a nationwide network that would involve medical professionals and relevant institutions/authorities such as government health authorities, qualified radiation protection professionals, e.g., medical physicists/radiation safety officers (RSOs) of cancer hospitals. This would contribute significantly to the proper monitoring of medical X-ray installations all over the country and improve radiological safety standards.

This audit revealed that the state of awareness regarding radiological safety, even among the radiologists in the country, is far from satisfactory. Among general physicians, this awareness is still less and often nonexistent. In view of this and because there are a large number of medical X-ray diagnostic installations in the country there is an urgent need of organising periodic nationwide awareness programmes by relevant medical professional bodies on radiation safety and regulatory aspects applicable to medical X-ray diagnostic practice. The findings of this radiation safety and QA audit provides valuable inputs for organizing such awareness programmes.

The outcome of this study also provides vital feedback, especially to make sufficient provisions with respect of QA and other radiological safety aspects, while revising the existing national safety code and also helps in the preparation of other documents such as a safety guide and quality criteria guidelines in X-ray diagnostic practice

The study thus contributed significantly in improving the radiological safety status of major medical X-ray diagnostic installations in the country.

## Conclusions

The radiological safety and QA audit of 118 medical X-ray diagnostic installations in India revealed several instances of noncompliance with the requirements of the national safety code; these were addressed and corrected. Some of the major noncompliances were: inaccuracy of kVp calibration (23%); noncongruence of radiation and optical field (23%); non linearity of mA station (16%) and timer (9%); faulty adjustor knob for alignment of field size (4%); improper collimator/diaphragm (19.6%); nonavailability of warning light (red light) outside X-ray room (29%); use of mobile protective barriers without lead glass viewing window (14%), etc. This survey generated awareness about the need and importance of in-house periodic radiological safety and QA audits to ensure optimimal radiological protection for patients, occupational workers in diagnostic radiology, and the public. This study makes significant contributions for improving the radiological safety status of medical X-ray installations in India and could provide a vital feedback in reviewing and preparation of regulatory documents pertaining to medical diagnostic X-ray practice.
